# Conservation of Avian Diversity in the Sierra Nevada: Moving beyond a Single-Species Management Focus

**DOI:** 10.1371/journal.pone.0063088

**Published:** 2013-05-07

**Authors:** Angela M. White, Elise F. Zipkin, Patricia N. Manley, Matthew D. Schlesinger

**Affiliations:** 1 Conservation of Biodiversity Group, Pacific Southwest Research Station, USDA Forest Service, Davis, California, United States of America; 2 USGS Patuxent Wildlife Research Center, Laurel, Maryland, United States of America; 3 Department of Biology, University of Maryland, College Park, Maryland, United States of America; 4 Conservation of Biodiversity Group, Pacific Southwest Research Station, USDA Forest Service, Hilo, Hawaii, United States of America; 5 Department of Environmental Science and Policy and Graduate Group in Ecology, University of California Davis, Davis, California, United States of America; 6 New York Natural Heritage Program, Albany, New York, United States of America; University of Western Ontario, Canada

## Abstract

**Background:**

As a result of past practices, many of the dry coniferous forests of the western United States contain dense, even-aged stands with uncharacteristically high levels of litter and downed woody debris. These changes to the forest have received considerable attention as they elevate concerns regarding the outcome of wildland fire. However, attempts to reduce biomass through fuel reduction (i.e., thinning of trees) are often opposed by public interest groups whose objectives include maintaining habitat for species of concern such as the spotted owl, *Strix occidentalis*, the northern goshawk, *Accipiter gentilis,* and the Pacific fisher, *Martes pennanti*. Whether protection of these upper-trophic level species confers adequate conservation of avian forest diversity is unknown.

**Methodology and Principal Findings:**

We use a multi-species occurrence model to estimate the habitat associations of 47 avian species detected at 742 sampling locations within an 880-km^2^ area in the Sierra Nevada. Our approach, which accounts for variations in detectability of species, estimates occurrence probabilities of all species in a community by linking species occurrence models into one hierarchical community model, thus improving inferences on all species, especially those that are rare or observed infrequently. We address how the avian community is influenced by covariates related to canopy cover, tree size and shrub cover while accounting for the impacts of abiotic variables known to affect species distributions.

**Conclusions and Significance:**

Environmental parameters estimated through our approach emphasize the importance of within and between stand-level heterogeneity in meeting biodiversity objectives and suggests that many avian species would increase under more open canopy habitat conditions than those favored by umbrella species of high conservation concern. Our results suggest that a more integrated approach that emphasizes maintaining a diversity of habitats across environmental gradients and minimizing urbanization may have a greater benefit to ecosystem functioning then a single-species management focus.

## Introduction

Biodiversity is integral to ecosystem functioning [1], [2], [3] and services that are essential for human well-being [4], [5]. Although the importance of biodiversity conservation is recognized, it remains one of the key challenges of land stewardship [6], [7], [8] due to several ecological and practical limitations [9]. First, the distribution, abundance and habitat requirements of all species in a particular area are rarely known, rendering optimal management solutions (if they exist) nearly impossible to discern. Second, management interventions that improve habitat conditions for one species can decrease the quality of habitat for others [10]. Lastly, how a species responds to a particular set of habitat conditions may vary spatially and temporally based on site-specific biotic and abiotic factors. These complexities, along with limited financial resources to monitor the impact of various management practices, have resulted in a reliance on single-species measurements and the hope that individual species can serve as indicators for the response of other species in the community [11].

Forested ecosystems support over 80% of terrestrial biodiversity worldwide and have high species diversity for many taxonomic groups, including birds [12], [13]. Although many of the earth’s forested landscapes are being lost due to conversion to agriculture and urban development, large stand-replacing fires are of additional concern in forested ecosystems in the mixed dry conifer forests of the western United States [14]. Prior to European influence these fire-adapted systems were structurally variable, with tree clusters of different size classes interspersed with open gap conditions [15], [16], [17]. However, over the past 100 years actions such as logging [18], [19], livestock grazing [20], [21], reduced burning practices by Native Americans [22], and fire suppression [23] have resulted in markedly different contemporary conifer forests [17], [24], [25]. These modifications have resulted in a decrease in forest structural variability at the stand and landscape levels. The forest is now characterized by high density, even-aged stands of more shade tolerant species such as firs and an understory with overall lower levels of shrub cover and higher amounts of downed woody material. Much attention from the management community has been placed on the increased risk of high-severity fire associated with changes in forest structure and composition [26]; however, these changes have also altered resource availability for forest-associated wildlife species with potentially substantial consequences for species occurrence and community structure. Managing these uncharacteristically high fuel loads in dry coniferous forests throughout the western United States dominate discussions of public forest land management policy [23], [27], [28].

Fuel reduction treatments are increasingly being applied in an attempt to reduce the risk of high-severity fire and increase forest resiliency [27], [29], [30]. Fuel reduction treatments are primarily designed to decrease fire intensity and the probability of crown fire through a reduction of fuels on the forest floor, increased height to the live crown, decreased crown density, and reduced densities of fire prone tree species [27]. Although many studies have demonstrated that fuel reduction treatments reduce the risk of high-severity wildfires [31], [32], there is continued public concern regarding the potential effects of these treatments on wildlife. Much of the conflict regarding forest management practices surrounds their potential impact on habitat for several species of concern, such as the spotted owl, *Strix occidentalis* [33], [34], the Pacific fisher, *Martes pennanti* [35], [36] and the northern goshawk, *Accipiter gentilis* [37]. In dry conifer forests in particular, the California spotted owl, *Strix occidentalis occidentalis,* and Pacific fisher are typically associated with denser, closed-canopy, multi-storied stands. Modification of these habitats to reduce the threat of fire is perceived to decrease habitat suitability for these old-growth forest associated species and fuel treatments in the Sierra Nevada are required to make special considerations for them. For instance, stands within spotted owl protected activity centers around known nests are required to be managed to maintain a minimum of 70 percent tree canopy cover and to retain dominant and co-dominant trees that are at least an average of 61 cm in diameter at breast height (DBH) [38]. Although suitable habitat for old-forest associates can span a range of characteristics, in general terms old forests in montane ecosystems of the Sierra Nevada are characterized as having a minimum of 60 percent tree canopy cover and minimum of 61 cm average DBH [39], [40].

Conservation of upper-trophic level species, such as the spotted owl, is thought to provide an umbrella of protection to other species that have similar habitat associations but have smaller area requirements [41], [42]. Land management planning commonly focuses on a few key species as a composite function of regulatory requirements, parsimony, and cost efficiency. The concept that management directed at one or a few species can meet the needs of the full suite of species with similar habitat associations is attractive, but the utility of this concept as a conservation tool has been questioned [43]–[46]. Managing for multiple species and subsequently validating outcomes pose many challenges [47], including the difficulty of estimating the abundances of all species within a community (or even a large subset). For instance, adjusting count data for observational bias in large-scale multi-species surveys is often difficult because a single adjustment method (i.e., distance sampling, double-sampling, removal methods, etc.) will not perform well for all species [48]. Additionally, methods that estimate population densities that account for imperfect detection are often limited to the most abundant species for which there is sufficient sample size [49]. Although a selection of some manageable number of species (e.g., N = 10) may help strengthen inferences about biodiversity, it is still likely to suffer from some of the same pitfalls as a single-species focus.

**Figure 1:**
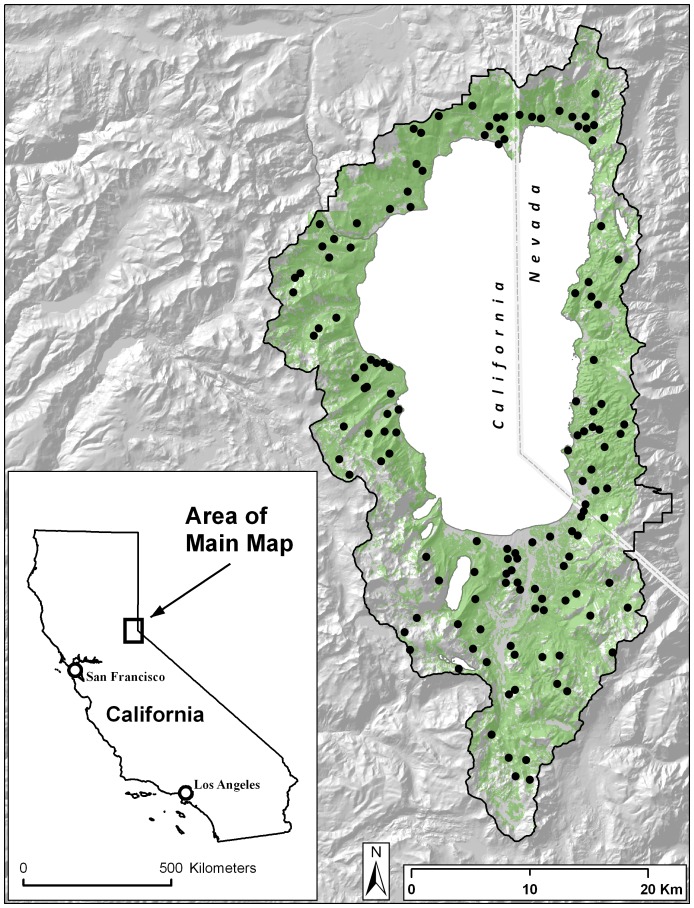
Location of the study area in California/Nevada, USA with 172 primary sample points indicated. Sampling points were distributed across forested areas (green) using systematic random sampling. Cluster sampling was conducted within 200 m of each primary sample point. Each of these 742 sites was sampled multiple times within a year for a total 2021 data points over the four-year study.

In this study, we use a multi-species occurrence model to address the habitat associations of an avian community in an 880-km^2^ conservation area, the Lake Tahoe Basin, located in the central Sierra Nevada Mountains (Figure 1). Our approach estimates the occurrence probability of all species in a community by linking multiple single-species occurrence models into a single model, thus improving inferences on all species, especially those that are rare or observed infrequently [50], [51], [52]. The purpose of this study is to understand the relationship between forest structural variables and the probability of species occurrences and to make inferences regarding the impacts that a management focus on umbrella species may have on conserving avian diversity. We address this question by modeling how the avian community is influenced by covariates related to canopy cover, tree size and shrub cover while accounting for other abiotic variables known to impact species distributions (elevation, precipitation and urban development). In light of our results, we discuss how future management strategies may better serve diversity-focused conservation objectives.

## Methods

### Study Area

The Lake Tahoe Basin is located on the eastern crest of the Sierra Nevada straddling the states of California and Nevada (Figure 1). Elevation and precipitation vary markedly in the basin and both environmental gradients are known to have large effects on productivity [53]. Elevation ranges from 1900 m at the surface of Lake Tahoe to 3400 m at the highest mountain peak. Mean annual precipitation is 150 cm, varying greatly with elevation and latitude, with the west shore experiencing 50% higher precipitation than the east shore [54]. The majority of precipitation occurs as snow and falls between the months of December and March. Approximately 67% of Basin’s forests were clear-cut during the last third of the 19^th^ century with less intensive harvesting continuing into the 20^th^ century [55]. Barbour *et al*. [15] suggested that less than 3% of the land in the Tahoe Basin remains unmodified since European influence in the early 1800’s. Common tree species today include Jeffrey pine (*Pinus jeffreyi*), white fir (*Abies concolor*), red fir (*A. magnifica*), incense-cedar (*Calocedrus decurrens*), lodgepole pine (*P. contorta*), and sugar pine (*P. lambertiana*).

### Avian Sampling

Avian point counts were conducted at 742 locations in the upland forested areas of the Lake Tahoe Basin Management Unit during the course of the breeding season (mid-May to early July) of 2002 to 2005 [56], [57]. Data from point counts used in this analysis came from two separate studies investigating wildlife-habitat relationships conducted over the same time period [58], [59]. Both studies used similar point count protocols in which all birds detected (seen or heard) in a 10-minute period within 100 m from the sample location were recorded. The majority of sites (72%) were sampled three times during the course of a breeding season, with the remaining sites limited to two sampling occasions due to logistical constraints. Visits to the same location were separated by approximately one week. Within a season, stations were visited by multiple observers (2 to 3 each year) to limit observer bias across study sites. Although locations were visited repeatedly within a season, each station was only visited in a single year. Point counts were located on federal, state and private lands. Although no formal permits were required, we received written or verbal permission from the corresponding landowners to sample at all sites.

Sample locations were selected using a combination of systematic/grid sampling and stratified random sampling. Four points were randomly selected from within a hexagonal grid laid across the Lake Tahoe Basin using spacing parameters of the Forest Inventory and Analysis program (N = 98). An additional 74 locations were randomly selected across a range of urban development classes. At each of these primary sampling locations, a cluster of additional sampling points was conducted 200 m from each primary point count station. Sampling locations for each year of the study were selected randomly. Combined, these sampling designs ensured that data points were distributed across the basin and adequately addressed the influence of urbanization. There was a minimum distance of 200 m between all sampling points.

### Habitat and Environmental Covariates

We characterized habitat using several explanatory variables with Geographic Information Systems (GIS) for the area within a 150-m radius of the survey locations. Although birds were recorded only when detected within 100 m of a sample point, we selected a 150-m radius to define our habitat variables as birds on the edge of the sampling radius are likely responding to surrounding forest conditions. Habitat parameters were derived from a GIS vegetation layer (30-m×30-m raster cell) based on IKONOS satellite imagery collected in 2002 [60]. Forest habitat parameters at our sample points included tree size (DBH) mean (range: 37–76 cm) and standard deviation (range: 0–12 cm), percent canopy cover mean (range: 2–66%) and standard deviation (range: 0–27%), and shrub cover mean (range: 9–66%). These parameters were selected to represent forest structure because they are components of the forest known to impact avian diversity [61], [62], [63] and these variables had low pairwise correlations (r ≤0.5) in our dataset. In addition, we extracted three environmental variables from remotely sensed databases that we hypothesized also would affect the probability of species occurrence: urban development, elevation and precipitation. The percentage of the area in urban land development was extracted from impervious surface data collected in 2003 and ranged in value form 0–79% across sample points [64]. Elevation (range: 1898–3160 m) was extracted from digital elevation maps and mean annual precipitation (range: 47–181 cm/yr) from 1997–2000 was used to interpret the gradient of variation in precipitation across the Basin [65].

### Data Analysis

We analyzed the data using a hierarchical multi-species modeling approach developed in Dorazio and Royle [66] and Dorazio et al. [50] to estimate species-specific occupancy probabilities relative to the abiotic and biotic (e.g., forest structure) variables. To do this, we combined individual species occurrence models in a single model by assuming that species covariate effects come from a common distribution, allowing for more precise estimates of occupancy [67], [68]. For species-level models, we assumed that the occurrence of species *i*, *z_i_*, is a Bernoulli process where the probability that species *i* is present at location *j* (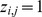 ) is 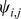 [69]. We then modeled the occurrence probability for each species *i* at location *j* using the logit link function and the relevant covariates such that:
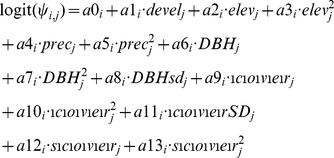
where 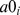 is the intercept and 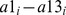 are the effects of the habitat covariates on species *i*. Parameters 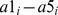 are the abiotic covariates that measure the effect of urban development (linear term), elevation (linear and squared terms), and precipitation (linear and squared terms) on the occurrence probability of species *i*. The parameters 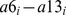 are effects related to the forest structure at location *j* (i.e., the forest within a 150-m radius of the point where the survey was conducted): 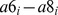 are the effects of the average diameter at breast height (DBH) of trees (linear and squared terms) and the standard deviation of DBH (linear term); 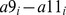 are the effects of percent forest canopy cover (linear and squared terms) and the standard deviation of forest canopy cover; 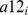 and 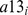 are the effects of percent forest shrub cover (linear and squared terms). We chose to include squared terms for some covariates, but not all, based on our hypotheses of the relationships of covariates to species occurrences. All covariates were standardized to have a mean of zero and a standard deviation of one.

Because species are detected imperfectly during sampling [69], we assumed that true occurrence, 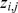, is a latent process that is only partially observable. If an observer detected species *i* at location *j* during sampling occasion *k*, denoted 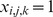, then it can be determined that 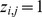. However, if a species is not detected it could be that the species was absent or that the species was missed during sampling. To account for detection biases, we used a repeated sampling protocol, assuming that the species pool was closed and that 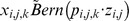 where 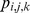 is the detection probability for species *i* at location *j* during sampling occasion *k* given that the species was present. We similarly modeled species detection probabilities using the logit link function:
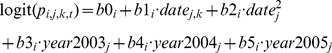
where 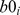 is the intercept, 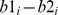 are the linear and squared effects of sampling day (Julian day 145–217, standardized to have mean zero and standard deviation of one) and 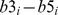 are year effects on detection as measured relative to a baseline year of 2002. Thus, our assumption is that the species pool was closed over the four-year sampling period (2002–2005) and similarly, that “occurrence” in this case is defined as species use of a location on at least one occasion during this time frame. We believe our occurrence estimates are robust to this assumption because each location was sampled in only one of the survey years and within a period of a few weeks. For the community-level component, we assumed that each of the species-specific parameter values from the occurrence (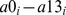) and detection (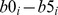) models were drawn from parameter-specific community-level distributions [50], [66]. Thus we assumed that each of the covariate estimates (e.g., all the 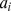 estimates) came from a normal distribution with a common mean and variance across all *i* species (e.g., 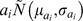).

Parameters were estimated using a Bayesian approach with Markov chain Monte Carlo (MCMC) implemented in the programs R and WinBUGS with flat priors for each of the community-level parameters. We ran three chains of the model for 15000 iterations after a burn-in of 10000 iterations and saved every fifth estimate (resulting in 1000 values for each parameter). We assessed that the model had convergence using the R-hat statistic [70] with max R-hat values less than 1.04 for all parameters. We did not perform a formal assessment of model fit to our data. Model assessment and selection is complex in hierarchical models in general, and in multi-species models in particular. As such, there is no well-established method to determine model fitness for community models [71]. However, because of the low correlations between our covariates and the number of nonzero parameter estimates (e.g., posterior intervals that did not overlap zero), we believe that our model is adequate in describing our data in that it balances the inclusion of relevant factors while maintaining parsimony relative to the amount of available data.

## Results

We recorded 61 species of birds during 2021 visits to 742 point count stations. Of these 61 species detected, we excluded five species from our models as they typically breed at lower elevations (Anna’s hummingbird *Calypte anna*, blue-gray gnatcatcher *Polioptila caerulea*, bushtit *Psaltriparus minimus*, lazuli bunting *Passerina amoena*, and orange-crowned warbler *Oreothlypis celata*) and were considered vagrants or non-breeding periodics (all sighted fewer than 10 times). The house finch *Carpodacus mexicanus* and mourning dove *Zenaida macroura* were also excluded from our analyses as their presence in forests is dependent upon urban development (i.e. they are not expected in forests lacking urbanization). These species were excluded from the model because they would not be representative of the community. Seven additional species considered very rare, Calliope hummingbird *Stellula calliope*, Hammond’s flycatcher *Empidonax hammondii*, lesser goldfinch *Spinus psaltria*, Pacific-slope flycatcher *Empidonax difficilis,* purple finch *Carpodacus purpureus*, ruby-crowned kinglet *Regulus calendula*, and yellow warbler *Dendroica petechia*), were observed fewer than 20 times, and they were included in our hierarchical model but not in our presentation of covariate estimates for individual species because their covariate estimates could be misleading. The mean and standard deviation of the occurrence and detection probabilities for all species included in the model are presented in Appendix S1. Mean covariate estimates for the occurrence model are presented for all species (except the seven very rare species) in Appendix S2.

Mean occurrence probabilities varied substantially across species from <2% to 98% (Appendix S1) when all abiotic and biotic covariates were held at their mean values. Urban development had the strongest, most consistent effect on the probability of species occurrence. The mean covariate estimate on urban development (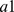) was negative for 37 species (indicating a decline in occupancy with higher levels of development for 80% of species) with the posterior intervals of 27 species not overlapping zero (Table 1). As predicted, avian species were also significantly (posterior interval did not overlap zero) influenced by elevation (29 species) and precipitation (9 species) with similar numbers of species responding positively and negatively to these parameters (Table 1).

**Table 1:** Number of species in which the species-specific parameter estimate was positive or negative.

Parameter	Positive	Negative
Development	10 (4)	37 (27)
Elevation	22 (8)	25 (13)
Elevation^2^	12 (0)	35 (13)
Precipitation	23 (11)	25 (10)
Precipitation^2^	22 (0)	25 (7)
Canopy cover	28 (10)	20 (4)
Canopy cover^2^	24 (0)	23 (3)
Canopy variance	21 (6)	26 (1)
Tree size (DBH)	23 (0)	24 (3)
Tree size (DBH)^2^	27 (2)	20 (0)
Tree size variance	24 (2)	23 (0)
Shrub cover	23 (7)	24 (4)
Shrub cover^2^	28 (1)	20 (1)

Values in parenthesis indicate the subset of species in which the posterior intervals do not overlap zero**.**

**Figure 2:**
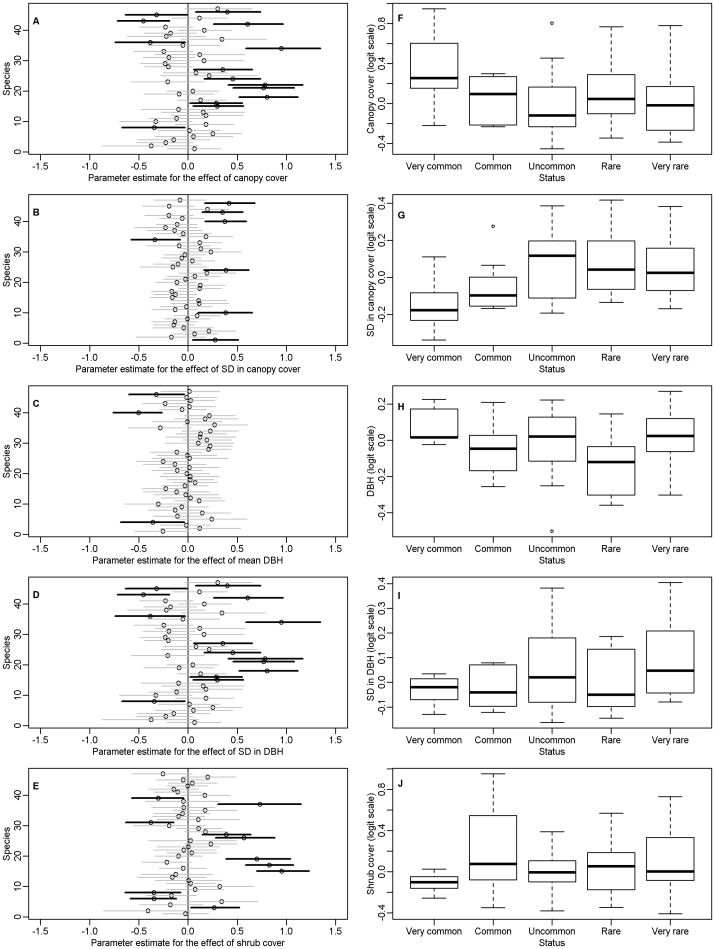
Mean parameter estimates and posterior intervals for the effect of A) percent canopy cover, B) standard deviation in canopy cover, C) mean DBH, D) standard deviation in DBH, E) percent shrub cover for each of the 47 species included in our analysis. Values indicate the change in occurrence predicted as a function of the change in one standard deviation of change in each response variable. For purposes of illustration, species were classified as very common (occupancy probability ≥85%, N = 6), common (≥50% and <85%, N = 8), uncommon (≥25% and <50%, N = 13), rare (≥10% and <25%, N = 8) and very rare (<10%, N = 11) based on their mean probability of occurrence for average environmental and habitat conditions in the Tahoe Basin (Appendix S1). Panels F-J show comparisons of the covariate estimates across these groups.

In general, mean parameter estimates for any single structural aspect of the forest were small relative to the abiotic variables suggesting that species within the basin may be more restricted by these factors than by variability in forest structure (Appendix S2). Of the modeled habitat covariates, percent canopy cover significantly influenced the occurrence probability of 16 species, DBH for five species and percent shrub cover for 13 species (Figure 2, Table 1). Increases in the standard deviation in canopy cover were associated with an increase in the probability of occurrence for six species and a decrease in occurrence probability for one species. Standard deviation in DBH affected fewer species, but higher variance was consistently associated with higher occurrence probability for two species (Figure 2, Table 1).

**Figure 3:**
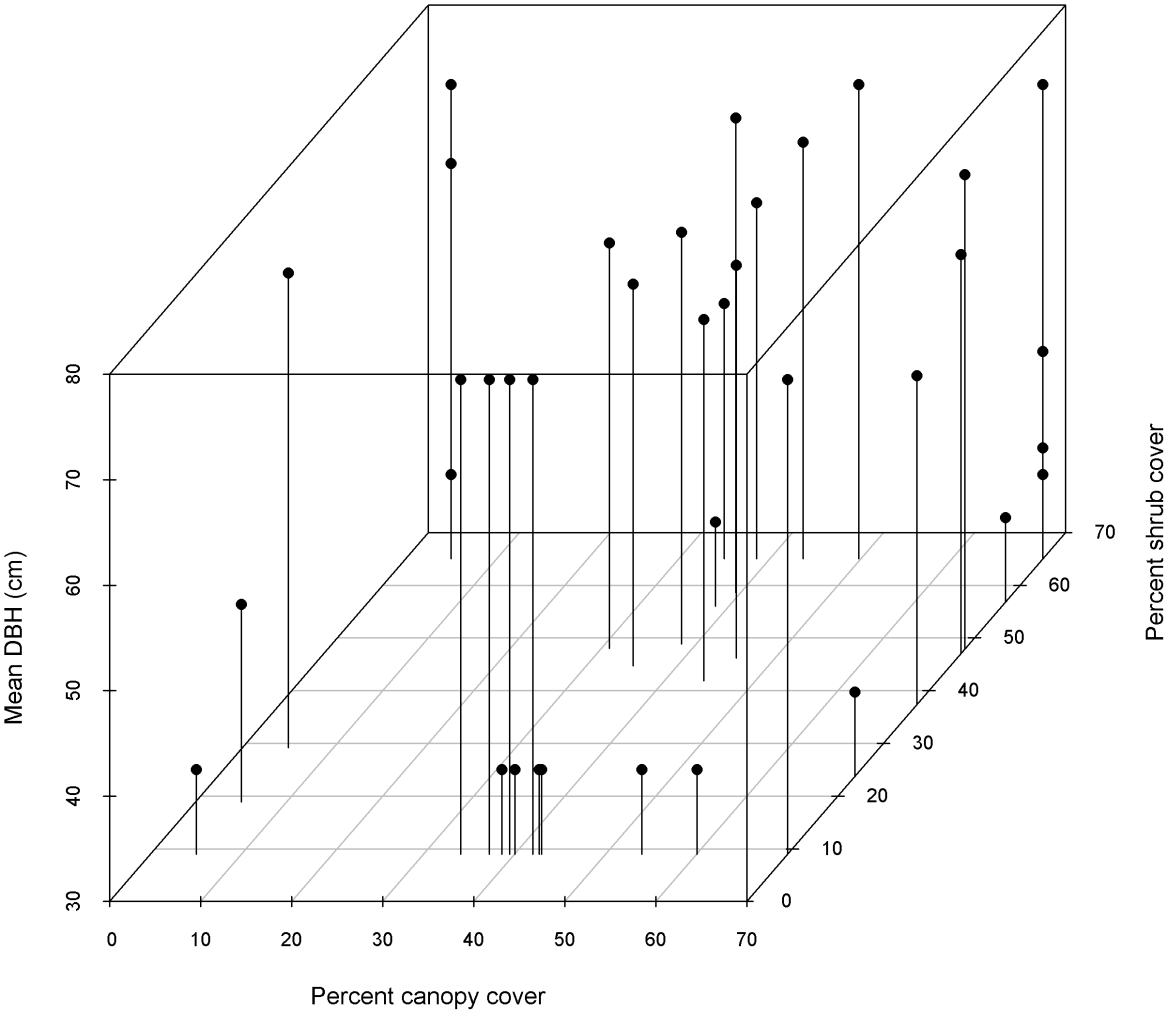
The intersection of covariate values for percent canopy cover (x-axis), mean tree DBH (y-axis), and percent shrub cover (z-axis) for 47 species included in our analysis. Each pinhead reflects the DBH value, while pin placement on the x-y surface indicates the percent canopy cover and percent shrub cover values that are predicted to result in the highest probability of occurrence for each species.

Variation in the response across species for each environmental variable underscores both the consistent effect of development and the importance of heterogeneous habitat for maintaining species diversity (Figure 2). Changes in forest structure associated with past management practices have typically led to a denser and more homogenized forest structure in both tree size and spacing. On average, many of these changes appear to have less impact on, or have led to an increase in, the more commonly occurring avian species (Figure 2). When investigating the combination of habitat variables that results in the highest probability of occurrence for each species we found that species-specific occurrence probabilities tended to be maximized within the range of 30–50 percent canopy cover, greater than 40 percent shrub cover and at the largest average tree sizes found within the basin (Figure 3). We also found that only six species reached their highest occurrence probability within the general domain of old forest conditions (minimum 60 percent tree canopy cover and minimum of 61 cm average DBH).

## Discussion

Effective conservation approaches and efficient management of forests are central biodiversity challenges. The results of our study suggest that a single-species-driven conservation approach, even one that targets a species that has the potential to serve an umbrella function, could lead to the prevalence of less suitable habitat for a sizeable percentage of the passerine birds using forested habitat in the Lake Tahoe Basin. For example, managing this landscape to maximize habitat for an old forest associate could decrease occurrence probabilities for nearly 90 percent of all species including the less commonly occurring species. Conversely, managing this landscape to maximize the probability of occurrence for the most species (i.e., maximizing species richness) is likely to decrease the occurrence probabilities for old-forest associates. Further, our results also underscore the consistently negative impact of urban development on many avian species in the Lake Tahoe Basin and suggest that actively managing the extent and placement of urban development to minimize biodiversity impacts is as important as considerations of forest structure.

Forest thinning projects have frequently been opposed by those concerned with maintaining suitable habitat for old-forest associated species of concern, such as the spotted owl, which require old forest conditions, namely high canopy cover and large trees [28], [33], [34]. Thinning of trees in fire-suppressed forests could increase the probability of occurrence of many avian species as gaps in the tree canopy have many beneficial ecological effects, including increasing plant diversity and shrub cover [72] and can provide a variety of microhabitat for shrub nesting birds and foliage gleaners [73]. Although fuel reduction projects using mechanical removal of trees may be able to open up the canopy quickly, shrub and tree growth will only occur over larger time scales. This is an important consideration as many of the modeled species had higher occupancy estimates associated with higher shrub cover and larger trees. As such, these species may respond negatively to fuel reduction treatments in the short term. However, several recent reviews have suggested that many vertebrate species respond neutrally or positively to fuel reduction treatments even within this timeframe [74], [75] suggesting that there may be immediate benefits unrelated to forest structure. For instance, avian species specializing on insects may benefit immediately from the opening of the canopy as light penetration and intensity can increase flying insect abundance and richness [76]–[79].

Parameters estimated through our multi-species model emphasize the importance of within and between stand-level heterogeneity in meeting biodiversity objectives. At the stand level, some species responded positively to higher variance in tree size and canopy cover; thus, increasing forest heterogeneity in forest stands would improve habitat suitability for these species (see [30], [80]). Species responses to the suite of abiotic and biotic variables were variable with a similar number of species in our models responding positively and negatively to the most influential abiotic and biotic variables, suggesting the importance of heterogeneity between forest stands. Additionally, both historical data and data from forested areas that have lacked fire suppression suggest that 30 percent shrub cover values were typical (e.g., [81]) and below the values of shrub cover that maximized occurrence probabilities for many species in our models. Many of these shrub cover values are also associated with canopy cover values that arguably would be too high to allow for the preferred level of shrub cover suggested by our results. This may reflect species, such as chipping sparrow *Spizella passerina*, that specialize on forest edge habitats and require both highly forested areas and shrub fields to meet their life history requirements. The results of our model indicate that past practices and management approaches that lead to increased homogenization of the forest will have negative impacts on avian diversity. Management approaches, such as fuel reduction treatments, or the use of prescribed or managed wildland fire, may be designed to restore at least some of the variability within and among stands that existed during an active fire regime, thereby enhancing habitat conditions for conserving avian biodiversity.

The link between habitat heterogeneity and biodiversity has been well-established (reviewed in [82]) and studies have shown that structurally complex landscapes can compensate for spatially restricted high-intensity management [83]. Structurally complex landscapes also increase resiliency and the capacity to recover from a disturbance [84]. Management actions that are driven by one or a few focal species do not appear adequate for maintaining avian biodiversity if their protection results in decreased variability in habitat conditions. An integrated approach that emphasizes conserving a diversity of habitats across environmental gradients and minimizing the extent of urbanization is likely to provide a greater benefit to conserving and restoring biodiversity and enhancing ecosystem functioning then a single-species focus. The use of multi-species approaches to inform land management could enhance biodiversity conservation by identifying habitat conditions that support unique suites of species. Management approaches that consider the extent and distribution of habitat conditions across landscapes have the greatest likelihood of conserving and restoring biodiversity and ecosystem functions.

## Supporting Information

Appendix S1 **Upland forest bird frequency status (ranging from very common to very rare) and mean detection and occupancy probabilities with one standard deviation.**
(DOCX)

Appendix S2 **Mean parameter estimates for upland bird species included in our analysis.** Values indicate the change in occurrence predicted for each change in one standard deviation in the response variable. Bold indicates that the posterior interval did not overlap zero. The following seven species were excluded from this table (but not the overall analysis) because they were observed fewer than 20 times: Calliope hummingbird *Stellula calliope,* Hammond’s flycatcher *Empidonax hammondii*, lesser goldfinch *Spinus psaltria*, Pacific-slope flycatcher *Empidonax difficilis,* purple finch *Carpodacus purpureus*, ruby-crowned kinglet *Regulus calendula*, and yellow warbler *Dendroica petechia*).
(DOCX)
