# Supplementary material for: Conservation of Avian Diversity in the Sierra Nevada: Moving beyond a Single-Species Management Focus
Source: PLoS One. 2013 May 7;8(5):e63088. doi: 10.1371/journal.pone.0063088 (PMC3646733; doi:10.1371/journal.pone.0063088)
Supplement: Appendix S1 — Upland forest bird frequency status (ranging from very common to very rare) and mean detection and occupancy probabilities with one standard deviation. (DOCX) [file pone.0063088.s001.docx]

| Species code | Common Name | Scientific Name | Status | Occurrence | Detection |
| --- | --- | --- | --- | --- | --- |
| AMRO | American robin | *Turdus migratorius* | Common | 0.85 ± 0.02 | 0.60 ± 0.02 |
| BBWO | Black-backed woodpecker | *Picoides arcticus* | Very rare | 0.05 ± 0.02 | 0.19 ± 0.06 |
| BHCO | Brown-headed cowbird | *Molothrus ater* | Common | 0.51 ± 0.05 | 0.67 ± 0.03 |
| BHGR | Black-headed grosbeak | *Pheucticus melanocephalus* | Rare | 0.11 ± 0.02 | 0.42 ± 0.06 |
| BRCR | Brown creeper | *Certhia americana* | Common | 0.72 ± 0.04 | 0.47 ± 0.02 |
| BTPI | Band-tailed pigeon | *Patagioenas fasciata* | Rare | 0.21 ± 0.03 | 0.50 ± 0.05 |
| CAFI | Cassin's finch | *Carpodacus cassinii* | Rare | 0.22 ± 0.04 | 0.34 ± 0.03 |
| CAHU* | Calliope hummingbird | *Stellula calliope* | Very rare | 0.01 ± 0.01 | 0.36 ± 0.10 |
| CAVI | Cassin's vireo | *Vireo cassinii* | Rare | 0.11 ± 0.02 | 0.38 ± 0.04 |
| CHSP | Chipping sparrow | *Spizella passerina* | Rare | 0.10 ± 0.02 | 0.36 ± 0.06 |
| CLNU | Clark's nutcracker | *Nucifraga columbiana* | Uncommon | 0.28 ± 0.04 | 0.44 ± 0.03 |
| CORA | Common raven | *Corvus corax* | Very rare | 0.07 ± 0.02 | 0.47 ± 0.06 |
| DEJU | Dark-eyed junco | *Junco hyemalis* | Very common | 0.96 ± 0.01 | 0.74 ± 0.02 |
| DOWO | Downy woodpecker | *Picoides pubescens* | Very rare | 0.07 ± 0.02 | 0.38 ± 0.06 |
| DUFL | Dusky flycatcher | *Empidonax oberholseri* | Common | 0.51 ± 0.05 | 0.56 ± 0.03 |
| EVGR | Evening grosbeak | *Coccothraustes vespertinus* | Common | 0.54 ± 0.04 | 0.50 ± 0.03 |
| FOSP | Fox sparrow | *Passerella iliaca* | Common | 0.77 ± 0.03 | 0.74 ± 0.02 |
| GCKI | Golden-crowned kinglet | *Regulus satrapa* | Uncommon | 0.44 ± 0.04 | 0.47 ± 0.03 |
| GTTO | Green-tailed towhee | *Pipilo chlorurus* | Very rare | 0.09 ± 0.02 | 0.34 ± 0.05 |
| HAFL* | Hammond's flycatcher | *Empidonax hammondii* | Very rare | 0.01 ± 0.00 | 0.38 ± 0.11 |
| HAWO | Hairy woodpecker | *Picoides villosus* | Uncommon | 0.40 ± 0.04 | 0.39 ± 0.03 |
| HETH | Hermit thrush | *Catharus guttatus* | Rare | 0.23 ± 0.04 | 0.49 ± 0.04 |
| HEWA | Hermit warbler | *Dendroica occidentalis* | Very rare | 0.07 ± 0.02 | 0.40 ± 0.05 |
| HOWR | House wren | *Troglodytes aedon* | Very rare | 0.05 ± 0.01 | 0.32 ± 0.05 |
| LEGO* | Lesser goldfinch | *Spinus psaltria* | Very rare | 0.00 ± 0.00 | 0.50 ± 0.15 |
| MGWA | Macgillivray's warbler | *Oporornis tolmiei* | Uncommon | 0.27 ± 0.04 | 0.37 ± 0.03 |
| MOCH | Mountain chickadee | *Poecile gambeli* | Very common | 0.98 ± 0.01 | 0.81 ± 0.02 |
| MOQU | Mountain quail | *Oreortyx pictus* | Rare | 0.22 ± 0.04 | 0.35 ± 0.04 |
| NAWA | Nashville warbler | *Oreothlypis ruficapilla* | Uncommon | 0.36 ± 0.05 | 0.55 ± 0.03 |
| NOFL | Northern flicker | *Colaptes auratus* | Common | 0.53 ± 0.04 | 0.45 ± 0.03 |
| OSFL | Olive-sided flycatcher | *Contopus cooperi* | Uncommon | 0.47 ± 0.04 | 0.47 ± 0.03 |
| PIGR | Pine grosbeak | *Pinicola enucleator* | Very rare | 0.07 ± 0.02 | 0.22 ± 0.05 |
| PISI | Pine siskin | *Spinus pinus* | Uncommon | 0.36 ± 0.04 | 0.43 ± 0.03 |
| PIWO | Pileated woodpecker | *Dryocopus pileatus* | Very rare | 0.03 ± 0.01 | 0.44 ± 0.08 |
| PSFL* | Pacific-slope flycatcher | *Empidonax difficilis* | Very rare | 0.01 ± 0.00 | 0.40 ± 0.12 |
| PUFI* | Purple finch | *Carpodacus purpureus* | Very rare | 0.01 ± 0.00 | 0.46 ± 0.12 |
| PYNU | Pygmy nuthatch | *Sitta pygmaea* | Uncommon | 0.29 ± 0.04 | 0.53 ± 0.04 |
| RBNU | Red-breasted nuthatch | *Sitta canadensis* | Very common | 0.92 ± 0.02 | 0.60 ± 0.02 |
| RBSA | Red-breasted sapsucker | *Sphyrapicus ruber* | Rare | 0.13 ± 0.03 | 0.32 ± 0.05 |
| RCKI* | Ruby-crowned kinglet | *Regulus calendula* | Very rare | 0.01 ± 0.00 | 0.34 ± 0.11 |
| RECR | Red crossbill | *Loxia curvirostra* | Very rare | 0.08 ± 0.02 | 0.25 ± 0.05 |
| SOGR | Sooty grouse | *Dendragapus fuliginosus* | Very rare | 0.03 ± 0.01 | 0.34 ± 0.07 |
| SPTO | Spotted towhee | *Pipilo maculatus* | Very rare | 0.02 ± 0.01 | 0.63 ± 0.06 |
| STJA | Steller's jay | *Cyanocitta stelleri* | Very common | 0.96 ± 0.01 | 0.74 ± 0.02 |
| TOSO | Townsend's solitaire | *Myadestes townsendi* | Uncommon | 0.45 ± 0.05 | 0.41 ± 0.03 |
| WAVI | Warbling vireo | *Vireo gilvus* | Uncommon | 0.38 ± 0.04 | 0.54 ± 0.03 |
| WBNU | White-breasted nuthatch | *Sitta carolinensis* | Common | 0.58 ± 0.05 | 0.35 ± 0.03 |
| WETA | Western tanager | *Piranga ludoviciana* | Very common | 0.89 ± 0.02 | 0.62 ± 0.02 |
| WEWP | Western wood-pewee | *Contopus sordidulus* | Uncommon | 0.49 ± 0.04 | 0.60 ± 0.03 |
| WHWO | White-headed woodpecker | *Picoides albolarvatus* | Uncommon | 0.32 ± 0.04 | 0.44 ± 0.04 |
| WISA | Williamson's sapsucker | *Sphyrapicus thyroideus* | Uncommon | 0.29 ± 0.05 | 0.24 ± 0.03 |
| WIWA | Wilson's warbler | *Wilsonia pusilla* | Rare | 0.15 ± 0.03 | 0.41 ± 0.04 |
| YRWA | Yellow-rumped warbler | *Dendroica coronata* | Very common | 0.92 ± 0.02 | 0.66 ± 0.02 |
| YWAR* | Yellow warbler | *Dendroica petechia* | Very rare | 0.02 ± 0.01 0.01 | 0.42 ± 0.10 |

*These species were included in the hierarchical model, but were not represented in the manuscript as they were detected too infrequently (N < 20) to produce precise parameter estimates.
