# Supplementary material for: Conservation of Avian Diversity in the Sierra Nevada: Moving beyond a Single-Species Management Focus
Source: PLoS One. 2013 May 7;8(5):e63088. doi: 10.1371/journal.pone.0063088 (PMC3646733; doi:10.1371/journal.pone.0063088)
Supplement: Appendix S2 — Mean parameter estimates for upland bird species included in our analysis. Values indicate the change in occurrence predicted for each change in one standard deviation in the response variable. Bold indicates that the posterior interval did not overlap zero. The following seven species were excluded from this table (but not the overall analysis) because they were observed fewer than 20 times: Calliope hummingbird Stellula calliope, Hammond’s flycatcher Empidonax hammondii, lesser goldfinch Spinus psaltria, Pacific-slope flycatcher Empidonax difficilis, purple finch Carpodacus purpureus, ruby-crowned kinglet Regulus calendula, and yellow warbler Dendroica petechia). (DOCX) [file pone.0063088.s002.docx]

| Species | Intercept | Develop | Elevation | Elevation^2^ | Precip | Precip^2^ | DBH | DBH^2^ | DBH SD | Canopy | Canopy^2^ | Canopy SD | Shrub | Shrub^2^ |
| --- | --- | --- | --- | --- | --- | --- | --- | --- | --- | --- | --- | --- | --- | --- |
| AMRO | **1.721** | 0.150 | -0.158 | -0.021 | -0.290 | 0.087 | -0.257 | 0.029 | 0.070 | 0.065 | -0.128 | **0.275** | -0.026 | 0.014 |
| BBWO | **-3.112** | **-0.798** | **0.751** | **-0.307** | -0.277 | -0.324 | 0.118 | -0.078 | -0.049 | -0.377 | -0.009 | -0.169 | -0.409 | -0.017 |
| BHCO | 0.032 | **0.552** | **-1.318** | 0.106 | **-0.708** | 0.054 | -0.019 | 0.026 | -0.074 | -0.228 | 0.027 | 0.065 | **0.266** | 0.106 |
| BHGR | **-2.139** | -0.007 | -0.031 | -0.037 | **-0.408** | -0.216 | **-0.358** | 0.106 | 0.124 | -0.146 | **-0.192** | 0.209 | -0.180 | -0.016 |
| BRCR | **0.967** | **-0.709** | -0.233 | -0.125 | -0.092 | -0.110 | -0.108 | -0.009 | 0.072 | 0.253 | -0.048 | -0.146 | **-0.348** | -0.015 |
| BTPI | **-1.308** | **0.622** | 0.141 | -0.115 | -0.099 | -0.156 | 0.145 | 0.015 | -0.067 | 0.013 | -0.158 | -0.134 | -0.169 | 0.029 |
| CAFI | **-1.294** | **-0.368** | **0.588** | 0.148 | -0.115 | 0.154 | -0.131 | 0.024 | -0.118 | **-0.345** | 0.092 | -0.007 | **-0.347** | 0.081 |
| CAVI | **-2.086** | **-0.944** | **-0.702** | -0.039 | -0.139 | -0.030 | -0.064 | 0.133 | -0.034 | 0.182 | 0.155 | 0.092 | 0.072 | 0.017 |
| CHSP | **-2.243** | **-0.480** | -0.195 | -0.004 | 0.166 | -0.148 | -0.303 | -0.045 | -0.058 | -0.329 | 0.034 | **0.383** | 0.322 | -0.140 |
| CLNU | **-0.937** | **-0.274** | **1.154** | 0.071 | **-0.933** | -0.024 | 0.112 | 0.070 | 0.033 | -0.118 | 0.043 | -0.132 | 0.026 | **0.203** |
| CORA | **-2.541** | 0.065 | **-0.512** | 0.099 | **-0.608** | -0.056 | 0.024 | 0.064 | -0.079 | 0.180 | 0.001 | -0.018 | 0.003 | -0.031 |
| DEJU | **3.311** | **-0.373** | 0.408 | **-0.242** | 0.276 | 0.094 | -0.024 | -0.135 | -0.044 | 0.154 | 0.034 | 0.111 | -0.162 | -0.063 |
| DOWO | **-2.604** | -0.243 | -0.332 | -0.222 | -0.300 | 0.106 | -0.119 | -0.003 | 0.203 | -0.098 | -0.025 | 0.106 | -0.126 | -0.060 |
| DUFL | 0.052 | **-1.238** | 0.318 | -0.100 | **0.382** | **-0.298** | -0.229 | 0.102 | 0.079 | **0.298** | 0.053 | -0.161 | **0.950** | -0.097 |
| EVGR | 0.179 | 0.152 | **-1.266** | 0.090 | **1.161** | -0.140 | -0.033 | -0.045 | -0.122 | **0.287** | -0.011 | -0.129 | -0.051 | -0.060 |
| FOSP | **1.222** | -0.046 | -0.079 | **-0.253** | **0.567** | **-0.254** | 0.073 | -0.056 | -0.007 | 0.128 | 0.071 | -0.166 | **0.827** | 0.011 |
| GCKI | -0.255 | **-0.527** | 0.129 | **-0.249** | **0.612** | -0.131 | 0.021 | -0.083 | 0.191 | **0.803** | -0.050 | 0.119 | -0.217 | -0.003 |
| GTTO | **-2.384** | -0.322 | **0.763** | **-0.246** | -0.368 | 0.073 | 0.023 | -0.029 | 0.109 | -0.091 | 0.162 | 0.124 | **0.699** | 0.030 |
| HAWO | **-0.415** | **-0.641** | **-0.519** | 0.048 | -0.117 | -0.138 | -0.014 | 0.058 | 0.099 | 0.048 | -0.049 | -0.114 | -0.098 | -0.060 |
| HETH | **-1.219** | **-1.356** | **0.771** | **-0.239** | **0.441** | -0.078 | -0.111 | 0.128 | -0.079 | **0.768** | 0.063 | -0.026 | 0.036 | 0.023 |
| HEWA | **-2.650** | **-1.246** | -0.137 | -0.144 | **0.449** | **-0.238** | 0.015 | 0.065 | **0.404** | **0.782** | 0.112 | 0.067 | -0.046 | 0.077 |
| HOWR | **-2.964** | **-0.373** | -0.198 | -0.123 | -0.146 | 0.050 | -0.136 | 0.027 | 0.236 | -0.207 | 0.111 | 0.191 | 0.001 | 0.064 |
| MGWA | **-0.990** | **-0.626** | 0.142 | -0.172 | 0.145 | 0.041 | -0.252 | -0.043 | **0.382** | **0.456** | -0.042 | **0.385** | 0.231 | 0.082 |
| MOCH | **4.091** | 0.322 | -0.600 | 0.012 | -0.121 | 0.039 | 0.017 | 0.149 | 0.015 | 0.214 | 0.080 | -0.156 | 0.024 | 0.022 |
| MOQU | **-1.263** | **-1.038** | -0.327 | **-0.365** | **1.420** | -0.234 | -0.008 | 0.040 | 0.145 | 0.082 | 0.035 | -0.103 | **0.569** | 0.040 |
| NAWA | **-0.560** | **-0.466** | **-0.498** | **-0.470** | **1.165** | **-0.360** | -0.115 | 0.068 | 0.180 | **0.352** | 0.094 | 0.043 | **0.387** | 0.069 |
| NOFL | 0.133 | -0.055 | **-0.853** | 0.141 | -0.199 | 0.020 | 0.209 | 0.006 | -0.104 | -0.200 | -0.076 | -0.064 | 0.176 | 0.072 |
| OSFL | -0.115 | **-0.464** | 0.146 | **-0.353** | 0.032 | 0.059 | 0.222 | -0.063 | -0.080 | -0.233 | -0.079 | -0.034 | 0.107 | 0.096 |
| PIGR | **-2.575** | -0.639 | **1.175** | -0.234 | **0.591** | -0.174 | 0.103 | -0.054 | 0.005 | 0.161 | -0.040 | 0.231 | -0.192 | -0.027 |
| PISI | **-0.579** | -0.220 | 0.091 | -0.067 | 0.139 | 0.082 | 0.189 | **0.145** | -0.157 | -0.196 | 0.011 | 0.129 | **-0.379** | -0.007 |
| PIWO | **-3.665** | **-0.756** | **-0.700** | -0.226 | -0.133 | 0.068 | 0.121 | 0.080 | 0.076 | 0.118 | 0.044 | -0.090 | 0.103 | 0.049 |
| PYNU | **-0.884** | **0.266** | **-0.705** | 0.077 | **-1.100** | 0.177 | 0.128 | -0.034 | 0.006 | -0.248 | -0.115 | 0.117 | -0.096 | -0.068 |
| RBNU | **2.504** | **-0.303** | -0.002 | -0.132 | **0.477** | **-0.329** | 0.225 | 0.120 | 0.006 | **0.948** | -0.050 | **-0.337** | -0.057 | 0.103 |
| RBSA | **-1.938** | -0.134 | 0.388 | -0.104 | 0.237 | -0.143 | -0.282 | -0.035 | -0.145 | -0.054 | -0.097 | 0.183 | 0.172 | 0.003 |
| RECR | **-2.464** | -0.174 | -0.243 | -0.145 | 0.128 | 0.043 | 0.269 | 0.044 | -0.037 | **-0.385** | 0.086 | -0.051 | -0.043 | 0.026 |
| SOGR | **-3.433** | **-0.800** | 0.411 | -0.217 | 0.053 | 0.093 | 0.240 | 0.021 | 0.213 | 0.054 | 0.119 | -0.046 | 0.341 | 0.037 |
| SPTO | **-3.777** | 0.104 | **-1.478** | -0.232 | **-1.274** | 0.073 | -0.007 | -0.124 | 0.018 | 0.345 | 0.100 | -0.136 | **0.729** | 0.016 |
| STJA | **3.339** | 0.269 | **-1.248** | 0.015 | **-0.604** | 0.086 | 0.172 | 0.014 | -0.070 | -0.220 | -0.049 | -0.230 | -0.047 | 0.110 |
| TOSO | -0.215 | **-1.125** | **0.520** | **-0.253** | **-0.427** | -0.058 | 0.215 | 0.051 | 0.184 | -0.178 | -0.071 | -0.111 | **-0.304** | -0.005 |
| WAVI | **-0.512** | **-0.701** | 0.314 | **-0.270** | 0.056 | 0.030 | **-0.503** | 0.045 | 0.021 | 0.165 | -0.096 | **0.373** | 0.171 | -0.057 |
| WBNU | 0.323 | **-0.733** | 0.233 | 0.097 | **-0.545** | -0.040 | -0.061 | -0.061 | -0.091 | -0.231 | **-0.231** | -0.060 | -0.106 | 0.020 |
| WETA | **2.103** | **-0.422** | 0.130 | **-0.320** | -0.291 | -0.047 | 0.014 | -0.118 | -0.130 | **0.605** | 0.083 | -0.195 | -0.147 | -0.017 |
| WEWP | -0.047 | **-0.742** | **-0.975** | -0.071 | **-0.292** | 0.019 | -0.233 | **0.153** | -0.080 | **-0.453** | **-0.135** | **0.349** | -0.005 | -0.013 |
| WHWO | **-0.751** | **0.293** | **-0.504** | 0.018 | 0.157 | **-0.293** | 0.024 | -0.036 | -0.163 | 0.115 | 0.002 | 0.196 | 0.043 | 0.112 |
| WISA | **-0.916** | **-0.730** | **0.767** | -0.122 | 0.300 | **-0.330** | -0.017 | -0.101 | -0.105 | **-0.319** | -0.118 | -0.191 | -0.052 | **-0.158** |
| WIWA | **-1.791** | **-0.550** | 0.553 | **-0.346** | 0.262 | 0.018 | **-0.323** | 0.064 | 0.186 | **0.399** | 0.074 | **0.416** | 0.199 | 0.057 |
| YRWA | **2.430** | -0.242 | 0.333 | -0.026 | **0.529** | 0.046 | 0.015 | -0.002 | 0.034 | 0.300 | -0.070 | -0.083 | -0.257 | -0.090 |
